# Transcriptomic and metabolic analyses reveal the potential mechanism of increasing steroidal alkaloids in *Fritillaria hupehensis* through intercropping with *Magnolia officinalis*

**DOI:** 10.3389/fpls.2022.997868

**Published:** 2022-10-07

**Authors:** Yuanyuan Duan, Xiaohong Liu, Jiaqi Wu, Jingmao You, Fanfan Wang, Xiaoliang Guo, Tao Tang, Mingyan Liao, Jie Guo

**Affiliations:** ^1^ Key Laboratory of Biology and Cultivation of Chinese Herbal Medicines, Ministry of Agriculture and Rural Affairs, Institute of Chinese Herbel Medicines, Hubei Academy of Agricultural Sciences, Enshi, China; ^2^ Hubei Engineering Research Center of Under-forest Economy, Hubei Academy of Agricultural Sciences, Wuhan, China; ^3^ Hubei Engineering Research Center of Good Agricultural Practices (GAP) Production for Chinese Herbal Medicines, Institute of Chinese Herbel Medicines, Hubei Academy of Agricultural Sciences, Enshi, China; ^4^ Productivity Promotion Center of Enshi Tujia and Miao Autonomous Prefecture, Bureau of Science and Technology of Enshi Tujia and Miao Autonomous Prefecture, Enshi, China

**Keywords:** *Fritillaria hupehensis*, *Magnolia officinalis*, intercropping, monocropping, transcriptomic, metabolic, steroidal alkaloid biosynthesis

## Abstract

*Fritillaria hupehensis*, a well-known medicinal perennial herb, is used as an antitussive and an expectorant. Continuous cropping and monoculture cultivation usually negativly affect the growth of *F. hupehensis*. Compared with the monoculture system, the *F. hupehensis*-*Magnolia officinalis* intercropping system significantly increases the yield of *F. hupehensis*. However, changes in steroidal alkaloid metabolites (the most important bioactive components) and their molecular regulatory mechanisms in *F. hupehensis* intercropping system remain unclear. We performed comparative transcriptomic and metabolomic analyses of *F. hupehensis* bulbs grown in monocropping and intercropping systems. A total of 40 alkaloids were identified, including 26 steroidal alkaloids, 4 plumeranes, 3 phenolamines, 1 pyridine alkaloid, and 6 other alkaloids. The results showed that intercropping significantly increased the levels of peimine, peiminine, hupehenine, korseveridine, verticinone N-oxide, delafrine, tortifoline, pingbeinone, puqienine B, puqienine E, jervine, ussuriedine, hydroxymandelonitrile, N-feruloylputrescine, and N-benzylmethylene isomethylamine in *F. hupehensis*, but decreased the levels of indole, p-coumaroylputrescine, and N-benzylformamide. Transcriptome sequencing identified 11,466 differentially expressed unigenes in *F. hupehensis* under the intercropping system, of which 5,656 genes were up-regulated and 5,810 genes were down-regulated. We proposed a possible steroidal alkaloid biosynthesis pathway, in which 12 differentially expressed genes were identified. The higher expressions of these genes in the intercropping system positively correlated with the high accumulation of peimine, peiminine, and hupehenine, further validating our proposal. Moreover, the biological processes of oxidative phosphorylation and plant hormone signal transduction, cytochrome P450 enzymes, ATP-binding cassette transporters, and transcription factors may play pivotal roles in the regulation of steroidal alkaloid biosynthesis. This study revealed the underlying molecular mechanisms of intercropping in improving steroidal alkaloids in *F. hupehensis* at the transcriptome and metabolome levels. These findings provided a theoretical foundation for sustainable development of this ecological planting method.

## Introduction

*Fritillaria hupehensis* (Hsiao et K.C. Hsia), a perennial herb of the *Liliacea*e family, has been widely used as a traditional Chinese medicine for more than 2000 years in China (Zhang et al., 2008; Guo et al., 2021). Bulbs of *F. hupehensis*, kwown as Hubeibeimu in *the Pharmacopoeia of the People’s Republic of China*, have been used as antitussives and expectorants (Zhang et al., 2007). Although various components, such as terpenoids, steroidal saponins, glycosides, and phenylpropanoids, have been identified, alkaloids are the largest class of bioactive components in *F. hupehensis* (Guo et al., 2021; Wang et al., 2021). In recent years, the demand for *F. hupehensis* has increased drastically, and the export of Chinese medicinal materials made from *Fritillaria* has increased by 133% in five years (Wang et al., 2021). However, the over-exploitation of wild resources has led to an increased market dependency on artificial cultivation. Moreover, *F. hupehensis* monoculture is constrained by productivity and quality challenges associated with crude cultivation methods and continuous cropping, resulting in an imbalance between supply and demand for *F. hupehensis* (Wang et al., 2021).

Intercropping, a productive low-input sustainable agricultural system (Duan et al., 2021), is beneficial for addressing the problems of low yield and quality caused by monoculture. Many intercropping patterns, such as Chinese chestnut-tea (Wu et al., 2021), tea-soybean (Duan et al., 2021), and *Atractylodes lancea*-peanut (Li et al., 2014), have been found to increase crop yields and quality. *F. hupehensis* and *Magnolia officinalis* (Rehd.et Wils) intercropping is complementary in time and space, that is, their peak demand for resources occurs at different times (Kermah et al., 2017). In addition, the intercropping pattern of *F. hupehensis*-*M. officinalis* is well established and has been widely used in Hubei Province. Our previous study showed that this intercropping system improved the yield of *F. hupehensis* (Duan et al., 2019). It is noteworthy that the objective of intercropping systems in common crops is to increase crop yields (Sun et al., 2014; Peng et al., 2021), whereas the aim of intercropping patterns among medicinal plants is mainly to improve the production of their bioactive components (Peng et al., 2021). Previous studies have shown that peimine, peiminine, hupehenine, and peimisine are the main phytochemicals in *F. hupehensis* (Che et al., 2020; Liu et al., 2021). However, the roles and underlying mechanisms of the intercropping system (*F. hupehensis*-*M. officinalis*) in regulating steroidal alkaloid biosynthesis in *F. hupehensis* remains unclear.

RNA sequencing (RNA-Seq) has been widely used to understand the functional characterization and molecular mechanisms of metabolite biosynthesis (Zhou et al., 2020; Xiao et al., 2021; Feng et al., 2022). Additionally, RNA-Seq can be used to infer the expression profiles of genes involved in steroidal alkaloid biosynthesis in *Fritillaria* plants (Zhao et al., 2018; Kumar et al., 2021). In previous studies, several possible steroidal alkaloid biosynthetic pathways of *Fritillaria* were proposed (Zhao et al., 2018; Sharma, et al., 2021). However, the steroidal alkaloid biosynthesis pathway of *F. hupehensis* is still unknown, and the key genes involved in the regulation of the biosynthesis of major phytochemicals have not been functionally characterized.

In this study, ultra-performance liquid chromatography-tandem mass spectrometry (UPLC-MS/MS) was used to explore the differences in alkaloid metabolomics in *F. hupehensis* under monoculture and intercropping systems. Then, a transcriptomic analysis was performed to reveal the expression variation of key genes related to steroidal alkaloid biosynthesis in *F. hupehensis* under different cropping systems. Moreover, we addressed whether and how the biosynthesis was associated with plant hormone signal transduction and oxidative phosphorylation. Our results could help to better understand the potential mechanism of the intercropping system in improving steroidal alkaloids in *F. hupehensis*, and is beneficial for promoting the sustainable development of *F. hupehensis*-*M. officinalis* ecological intercropping systems.

## Methods and materials

### Plant materials and experimental design

**Figure 1:**
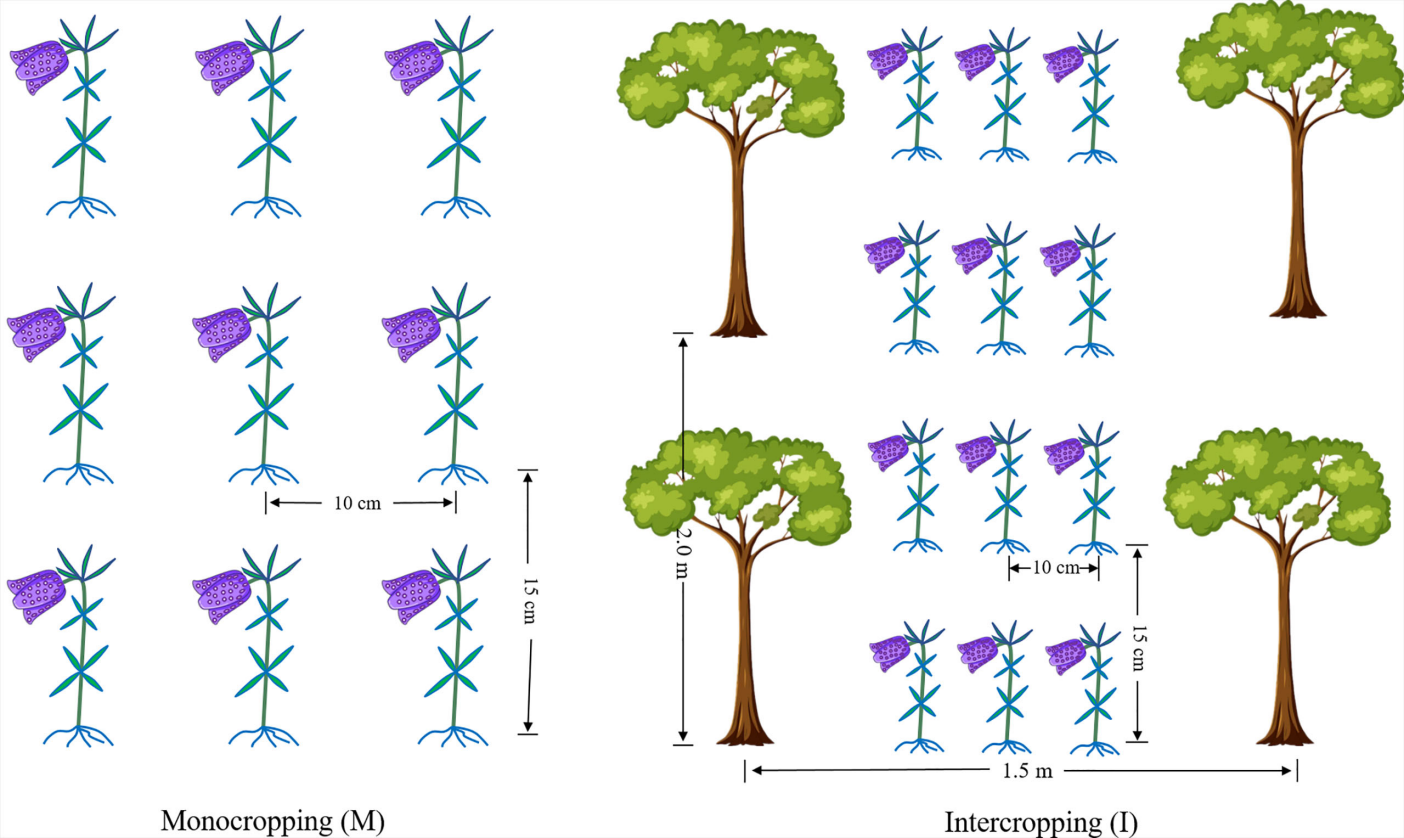
The cropping patterns of *F. hupehensis* monocropping and *F. hupehensis-M. officinalis* intercropping.

The experimental sites are located in Xintang Township, Enshi, Hubei Province, China (109°46′41″ E, 30°11′57″ N, altitude 1600 m). The average temperature was 16°C, the average relative humidity was 82%, the average annual precipitation was 1,500 mm, and the soil was yellow-brown earth with a sandy loam texture. Two cropping systems, *F. hupehensis* monocropping (M) and *F. hupehensis* intercropping with *M. officinalis* (I), were studied (**Figure 1**). One-year-old *M. officinalis* tree seedlings were planted in the year 1996, with a plant row spacing of 1.50 m × 2.00 m. By 2020, the trees were 3.50-4.00 m in height and 2.50-3.00 m in crown breadth. Fresh bulbs of *F. hupehensis* were graded before the test, and bulbs of the same size and weight (± 4 g) were selected as seeds (± 20% error). Fresh bulbs were sown with a row spacing of 10 cm × 15 cm in the I and M systems. The experiment was arranged in a completely random design with three replicates. The plot size (24 rows and 12 columns) was 6.67 m^2^ (1.50 m × 4.45 m). All plots were similarly maintained, under a conventional management model.

Regenerated fresh bulbs of *F. hupehensis* were collected from 9 a.m. to 10 a.m. on June 1, 2021. The samples collected from the I and M systems were designated I1, I2, and I3, and M1, M2, and M3, respectively. Each collected sample was divided, immediately frozen in liquid nitrogen, and stored at -80°C before RNA extraction or metabolite detection.

### Metabolic analysis

All samples were freeze-dried using a vacuum freeze-dryer (Scientz-100F, China) and then ground into a powder. For each sample, 100 mg of lyophilized powder was mixed with 1.20 mL of a 70% methanol solution. The extracts were vortexed six times, for 30 s every 30 min and then stored in a refrigerator at 4°C for 12 h. The extracts were then centrifuged at 12,000 r/min for 10 min and filtered through a 0.22 μm membrane filter (SCAA-104, ANPEL, China) before UPLC-MS/MS analysis.

Next, a UPLC (SHIMADZU Nexera X2, Japan)-electron spray ionization (ESI)-MS/MS (Applied Biosystems 4500 Q TRAP, USA) system was used to metabolize alkaloids. The UPLC column was Agilent SB-C18 (1.80 µm, 2.10 mm × 100 mm). The solvent phase consisted of water with 0.1% formic acid (A) and acetonitrile with 0.1% formic acid (B). A gradient program was performed with starting conditions of 5% B. Mobile phase B was increased to 95%, with a linear gradient, within 9 min, and maintained for 1 min. Subsequently, mobile phase B was adjusted to 5% within 1.10 min and maintained for 2.90 min. Samples were separated using a 40°C column at 0.35 mL/min, with an injection volume of 4 μL. The effluent was alternately connected to an ESI-triple quadrupole-linear ion trap (QTRAP)-MS. Mass spectrometry, metabolite identification, and quantification were performed following the standard procedures of Wuhan MetWare Biotechnology Co., Ltd., as described by Chen et al. (2020).

Analyst software (version 1.6.3, AB Sciex, USA) was used to process the mass spectral data. Orthogonal partial least squares-discriminant analysis (OPLS-DA) was used to check the variable importance in projection (VIP), and the data were log-transformed (log_2_) before OPLS-DA. Metabolites satisfying VIP≥1 and |log_2_ (fold change) |≥1 were defined as differential expressed metabolites (DEMs) between two groups. Thereafter, Kyoto Encyclopedia of Genes and Genomes (KEGG) enrichment analysis was performed.

### Transcriptomic analysis

Total RNA was extracted using the RNAprep Pure Plant Kit (Tiangen, China) according to the manufacturer’s instructions. RNA samples that met the quality requirements were used to construct a sequencing library and then sequenced on the Illumina HiSeq 6000 platform. Low-quality reads and reads containing adapters and poly-N were removed using fastp (version 0.19.3; Chen et al., 2018), and clean reads were *de novo* assembled using Trinity (version 2.11.0; Grabherr et al., 2011) to obtain high-quality transcript sequences. Fragments per kilobase of exon per million fragments mapped (FPKM) was used to estimate the gene expression levels. Subsequently, differential expression analysis between two groups was performed using the DESeq2 software (version 1.22.1; Love et al., 2014). Genes satisfying |Log_2_ (fold change) |≥1, false discovery rate (FDR), and adjusted *p* < 0.05 were defined as differentially expressed genes (DEGs). Then, Gene Ontology (GO), KEGG enrichment analysis and transcription factors (TFs) analysis were performed.

All raw sequencing data were submitted to the Genome Sequence Archive (GSA) database under the BioProject accession number CRA007535 at the BIG Sub website (https://ngdc.cncb.ac.cn/gsub/).

### qRT-PCR analysis

Nine DEGs were randomly selected for quantitative real-time polymerase chain reaction (qRT-PCR) with 18S rRNA as the reference gene. Specific primers were designed using Primer Premier 5.0, and the primer sequences were listed in **Table S1**. The qRT-PCR was run as follows: 95°C for 2 min, followed by 40 cycles at 95°C for 10 s, and 60°C for 30 s. The relative expression levels of the tested genes were calculated using 2^-ΔΔCt^. The value for each biological replicate was calculated using three technical replicates.

### Statistical analysis

Statistical analysis of the data was performed using Excel 2019 and SPSS 19.0. All data were expressed as mean ± standard deviation (n=3). One-way analysis of variance (ANOVA) was performed to examine differences between samples, and Duncan’s multiple range test (*p* < 0.05) was conducted to determine significant differences. The Pearson correlation (R-value) between DEGs and steroidal alkaloids was calculated. TBtools software (version 1.068) was used to generate heatmaps of DEGs and DEMs. Graphs were plotted using Origin Pro 2021 software.

## Results

### Alkaloid changes in response to the intercropping system

A total of 40 alkaloid metabolites were identified in the I and M systems, including 26 steroidal alkaloids, 4 plumerane, 3 phenolamine, 1pyridine alkaloid, and 6 other alkaloids (**Table S2**). The principal component analysis of UPLC-EDS-MS/MS data showed that all samples were divided into I and M groups, and three biological replicates of each system tended to group (**Figure S1A**). The Pearson correlation coefficients among biological replicates within a group were more than 0.98 (*p*<0.001), which were significantly higher than those among samples from different treatments (**Figure S1B**). Data reliability analysis revealed that our metabolomic data were highly replicable and qualified for further analysis (**Figure S1**).

**Figure 2:**
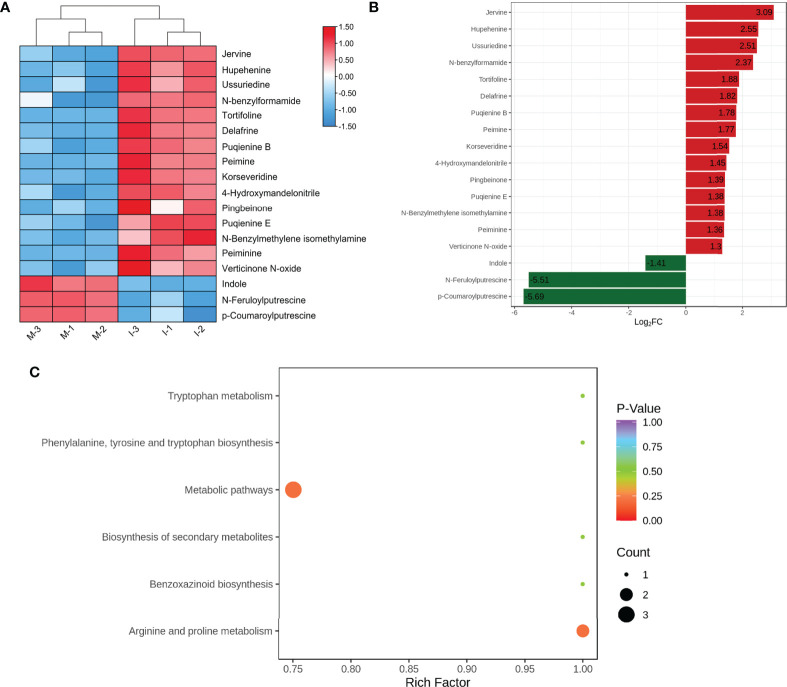
Analysis of DEMs in response to the intercropping system. **(A)** Heatmap of identified differential alkaloid metabolites. **(B)** Top fold change bar chart of identified DEMs. **(C)** Bubble map of metabolite pathway enrichment analysis in the I and M systems.

Based on OPLS-DA analysis, 18 significant DEMs were screened according to VIP ≥1 and |log_2_ (fold change) | ≥ 1(**Figure 2A**; **Table S3**). Compared with the M system, the top five significantly up-regulated DEMs (jervine, hupehenine, ussuriedine, N-feruloylputrescine, and tortifoline) were steroidal alkaloids (**Figure 2B**; **Table S3**). Furthermore, 12 identified DEMs, peimine, peiminine, hupehenine, korseveridine, verticinone N-oxide, delafrine, tortifoline, pingbeinone, puqienine B, puqienine E, jervine, and ussuriedine were up regulated in response to intercropping (**Figure 2A**). These results indicated that intercropping increased the levels of steroidal alkaloids in *F. hupehensis*. In addition, the levels of 4-hydroxymandelonitrile, N-feruloylputrescine, and N-benzylmethylene isomethylamine were significantly up-regulated in the I system, whereas indole, p-coumaroylputrescine and N-benzylformamide were down-regulated, indicating that intercropping also altered other alkaloids of *F. hupehensis*.

KEGG enrichment analysis was conducted to functionally classify DEMs from the I and M systems into different pathways. Compared with the M system, 6 alkaloid metabolites were related to metabolic pathways, but the *p*-value of the metabolic pathways was greater than 0.20, which showed no significant difference between the I and M systems (**Figure 2C**).

### Gene expression profiles in response to the intercropping system

An overview of the RNA-Seq results is presented in **Table S4**. In total, we identified 11,466 DEGs (5,656 up-regulated and 5,810 down-regulated) when comparing the I and M systems. GO enrichment analysis revealed that these DEGs were widely distributed among the three groups, “biological processes”, “cellular components”, and “molecular functions” (**Figure S2**). KEGG pathway annotation illustrated that “metabolic pathways” (1,662; 47.32%), “biosynthesis of secondary metabolites” (941; 26.79%), “plant-pathogen interaction” (228; 6.49%), “plant hormone signal transduction” (219; 6.24%), and “starch and sucrose metabolism” (201; 5.72%) were the top five subcategories (**Figure S3**).

**Figure 3:**
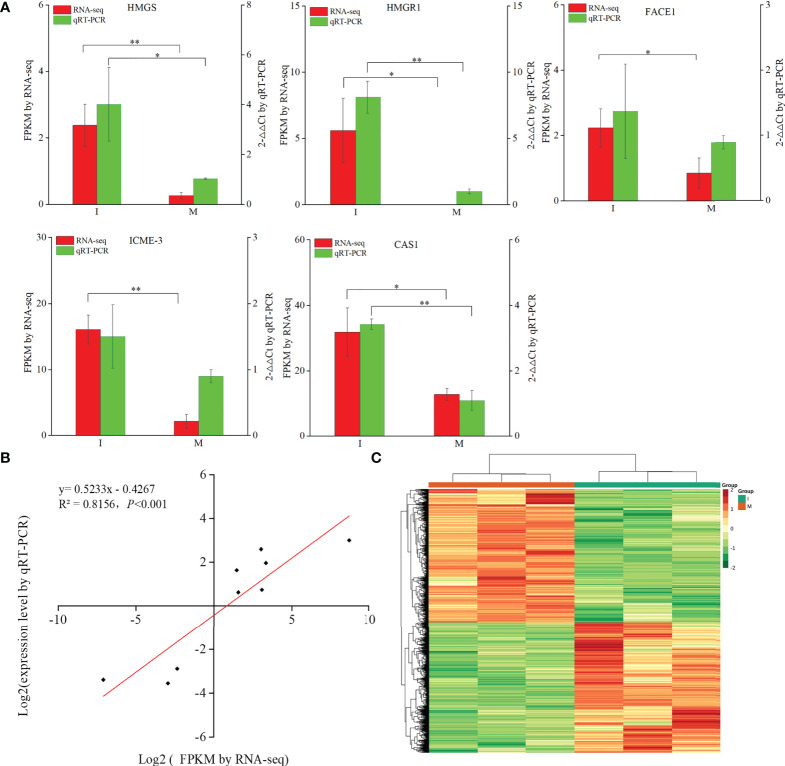
Analysis of DEGs replying to the intercropping system. **(A)** Comparison of the expression data of 5 key DEGs in steroidal alkaloids biosynthesis obtained by RNA-Seq and qRT-PCR. All data in the figure are indicated by mean ± SD (*, *p*<0.05; **, *p*<0.01). **(B)** Correlation of the results between RNA-Seq and qRT-PCR. The results were calculated by log_2_ fold change. **(C)** Hierarchical clustering of all the DEGs.

9 DEGs (6 up-regulated and 3 down-regulated) were randomly selected to confirm the reliability of the RNA-Seq results (**Table S5**). 5 genes related to steroidal alkaloid biosynthesis were selected to compare the expression data obtained by RNA-Seq and qRT-PCR (**Figure 3A**). Correlation analysis of gene expressions and the correlation coefficient (R^2^ = 0.8156, *p* < 0.0001) showed that our RNA-Seq results were reproducible (**Figure 3B**). Correlation heat map analysis was used to verify the reliability of the gene expression results (**Figure S4**). Furthermore, hierarchical clustering of the gene expression profiles showed that all DEGs were classified into two groups with opposite expression profiles between the I and M systems (**Figure 3C**).

### Differentially expressed genes in the steroidal alkaloid biosynthesis

**Figure 4:**
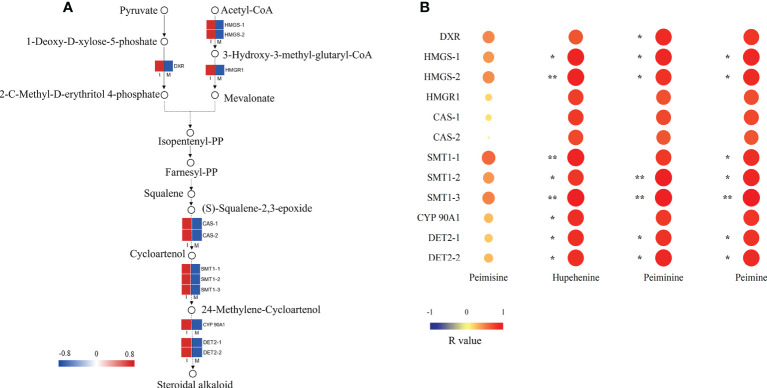
Key DEG analysis in steroid alkaloid biosynthesis. **(A)** Expression profiles of DEGs in the steroidal alkaloid biosynthesis pathway. Red color denoted genes with high expression level, while blue color denoted genes with low expression level. The relative expression levels of DEGs were calculated using log2 ratio. **(B)** Heat map of correlation between FPKM values of DEGs and main steroidal alkaloids, the correlation was indicated by Pearson r value (*p<0.05; **p<0.01).

A steroidal alkaloid biosynthesis pathway was proposed by analyzing the chemical structures of key intermediate compounds and their possible biocatalytic enzymes in the steroidal alkaloid biosynthesis of *F. hupehensis* (**Figure 4A**). 12 DEGs identified in this pathway were up-regulated. Interestingly, genes encoding 3-hydroxy-3-methylglutaryl-coenzyme A (HMG-CoA) synthase (HMGS), HMG-CoA reductase (HMGR), and 1-deoxy-D-xylulose-5-phosphate reductoisomerase (DXR), which are involved in the 2-C-methyl-d-erythritol 4-phosphate (MEP) and Mevalonate(MVA) pathways, were significantly enriched in the I system (**Figure 4A**; **Table S6**). In this study, 2 genes encoding cycloartenol synthase (CAS) involved in cycloartenol biosynthesis were up-regulated. Subsequently, the R-value between 12 DEGs and 4 main alkaloids (peimisine, peiminine, peimine, and hupehenine) were calculated (**Figure 4B**). The genes encoding HMGS, sterol C-methyltransferase 1 (SMT1), steroid 5-alpha-reductase DET2 (DET2), and steroidal alkaloids such as peiminine, peimine, and hupehenine were significantly positively correlated (*p* < 0.05; R-values>0.80). The result indicates that these genes may play an important role in the biosynthesis of the steroidal alkaloids. Moreover, the R-value between the genes encoding DXR and peiminine was 0.84 (*p* < 0.05), suggesting that *DXR* might be involved in peiminine biosynthesis. The R-value of the gene encoding cytochrome P450 (CYP) 90A1 and hupehenine was 0.83 (*p* < 0.05), suggesting that *CYP 90A1* might be involved in the biosynthesis of hupehenine.

### Differentially expressed genes involved in the oxidative phosphorylation and plant hormone signal transduction pathways.

**Figure 5:**
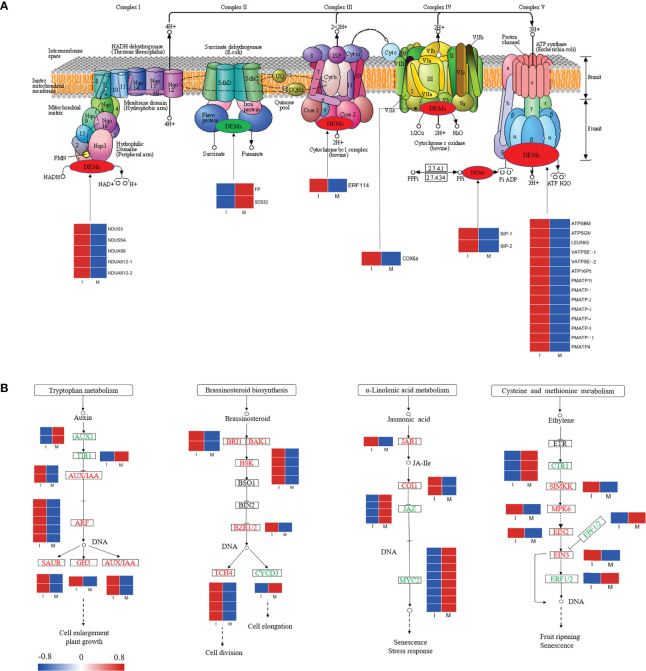
Expression profiles of DEGs in the oxidative phosphorylation pathway **(A)** and partial plant hormone signal transduction pathway **(B)**. Red color denoted genes with high expression level, while blue color denoted genes with low expression level. The relative expression levels of DEGs were calculated using log_2_ ratio.

In our study, 25 DEGs were identified in the comparison between the I and M systems, and most of the genes in the oxidative phosphorylation pathway were up regulated in the I system compared with the M system (**Figure 5A**; **Table S7**). Genes encoding NADH dehydrogenase (NDUS3, NDUS5A, NDUAS6, and NDUAS12), ATP synthase (ATPSBM, ATPSGM, LEUNIG, VATPSE1, ATP16PS, PMATP11, PMATP, and PMATP4), cytochrome c oxidase (COX6a), soluble inorganic pyrophosphatase (SIP), and ethylene-responsive transcription factor (ERF 114) were up-regulated. The results showed that intercropping might increase the expressions of these genes in *F. hupehensis*, which would accelerate oxidative phosphorylation metabolism and release more energy for steroidal alkaloid biosynthesis.

Variations in genes involved in partial plant hormone signal transduction pathways were analyzed (**Figure 5B**; **Table S8**). In this study, 15, 12, 15, and 9 DEGs were identified in the auxin, brassinosteroid, jasmonic acid (JA), and ethylene signaling pathways, respectively. Compared with the M system, 80.0% and 91.6% of the DEGs related to the auxin and brassinosteroid signaling pathways were up-regulated in the I system, respectively, whereas 80.0% and 55.5% of the DEGs associated with JA and ethylene signaling pathways were down-regulated, respectively. Compared with the I system, genes encoding MYC2, small auxin-up RNA (SAUR), Gretchen Hagen3 (GH3), and auxin/indole-3-acetic acid (AUX/IAA) were significantly up-regulated in the I system. The activation of the auxin, brassinosteroid, JA, and ethylene signaling pathways might initiate a series of physiological and biochemical reactions for steroidal alkaloid biosynthesis in *F. hupehensis* under the I system.

### Differentially expressed cytochrome P450s and ATP-binding cassette transporters related to biosynthesis of steroidal alkaloids

**Figure 6:**
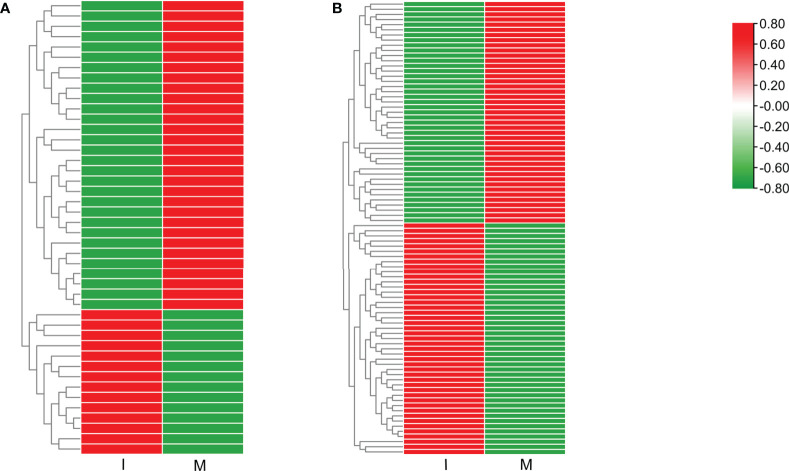
CYPs and ABC transporters involved in the synthesis of steroid alkaloids. **(A)** CYPs. **(B)** ABC transporters. Red color denoted genes with high expression level, while green color denoted genes with low expression level. The relative expression levels of DEGs were calculated using log_2_ ratio.

In this study, 44 genes encoding CYPs and 88 genes encoding ATP-binding cassette (ABC) transporters were identified in the *F. hupehensis* transcriptome. In the I system, 14 and 45genes encoding CYPs and ABC transporters were significantly up-regulated, respectively (**Figures 6A; B**, **Table S9**), while 30 and 43 genes encoding CYPs and ABC transporters were significantly down-regulated, respectively, compared with the M system, indicating that their expressions were complex in *F. hupehensis*.

### Transcription Factors related to the biosynthesis of steroidal alkaloids

**Figure 7:**
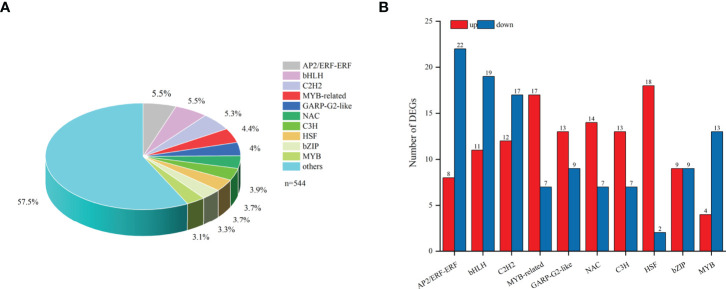
TFs DEGs involved in the synthesis of steroid alkaloids. **(A)** TFs. **(B)** Top10 TFs in samples under the I and M systems.

In total, 544 differentially expressed TFs were annotated in the transcriptome of *F. hupehensis* and classified into 44 families (**Table S10**). The top ten TF families were bHLH (5.5%), AP2/ERF-ERF (5.5%), C2H2 (5.3%), MYB-related (4.4%), GARP-G2-like (4%), NAC (3.9%), C3H (3.7%), HSF (3.7%), bZIP (3.3%), and MYB (3.1%) (**Figure 7A**). The results showed that most of the genes encoding MYB-related, GARP-G2-like, NAC, C3H, and HSF were up-regulated, while most of the genes encoding bHLH, AP2/ERF-ERF, C2H2, and MYB were down-regulated (**Figure 7B**).

## Discussion

UPLC-EDS-MS/MS is commonly used to identify secondary metabolites and bioactive components of Chinese herbal medicines (Chen et al., 2020; Xu et al., 2020). In this study, we used a UPLC-EDS-MS/MS approach to identify alkaloid metabolites in *F. hupehensis* under monocropping and intercropping systems. Most of the 40 identified alkaloid metabolites were steroidal alkaloids, indicating that steroidal alkaloids were the largest class of alkaloid metabolites in *F. hupehensis*. These results provided a global overview of alkaloid compounds and a reference for the future utilization of these alkaloids, in addition to confirming the effectiveness of UPLC-ESI-MS/MS for their identification. 12 differentially expressed steroidal alkaloids were more abundant in the intercropping system, which suggested that intercropping with *M. officinalis* significantly increased the accumulation of steroidal alkaloids in the bulbs of *F. hupehensis*. Peimine, peiminine, hupehenine, and peimisine are the main phytochemicals in *Fritillaria* plants (Che et al., 2020; Liu et al., 2021). In the present study, peimine, peiminine, and hupehenine were identified as positive DEMs, suggesting that the majority of the active components in *F. hupehensis* were higher in the I system than in the M system. In addition, intercropping significantly increased the levels of 4-hydroxymandelonitrile, N-feruloylputrescine, and N-benzylmethylene isomethylamine in *F. hupehensis*, while decreasing the levels of indole, p-coumaroylputrescine and N-benzylformamide. However, alkaloid metabolism in *F. hupehensis* was not significantly different between the I and M systems (**Figure 2C**), and further research should be conducted to verify these results.

Previous studies have shown that the MEP and MVA pathways are the main routes for steroid alkaloid biosynthesis in plants (Zhao et al., 2018; Wang et al., 2019), and cycloartenol serves as the major intermediate in the formation of steroid alkaloids (Kumar et al., 2021). Combined with our transcriptomic data, a steroidal alkaloid biosynthesis pathway was proposed, which mainly consisted of three parts: sterol C isoprene units synthesized by the MVA or MEP pathway, intermediates, and downstream biosynthesis of steroidal alkaloids. In the present study, 2 *HMGS* genes and 1 *HMGR* gene in the MVA pathway were significantly up-regulated in the I system compared with the M system, indicating more intermediates HMG-CoA and isopentenyl diphosphate (IPP) might be accumulated in *F. hupehensis* under the I system (Suzuki and Muranaka 2007; Liao et al., 2014; Feng et al., 2016; Zhou et al., 2019). Similarly, the rate-limiting enzyme gene *DXR* was over-expressed in the I system, which may promote the conversion of 1-deoxy-d-xylulose 5-phosphate (DXP) to MEP (Upadhyay et al., 2018; Kumar et al., 2020), leading to an increase in 2,3-oxidosqualene. Subsequently, 2,3-oxidosqualene is cyclized by the *CAS* genes to produce more cycloartenol, leading to further steroidal alkaloid biosynthesis through various modification reactions by CYPs, methyltransferases, reductase, and isomerase (Kumar et al., 2021). SMT1 acts at the initial step in the downstream pathway, catalyzing sterol biosynthesis *via* a single methyl addition at C-24 (Carland et al., 2010). In this study, the up-regulation of the three *SMT1* genes in the I system might accelerate the metabolism of SMT1, leading to an increase in the downstream products of the steroidal alkaloid biosynthetic pathway. CYP 90A1 has been reported to be responsible for C-23 hydroxylation of the steroid side chain (Kreis and Müller-Uri, 2010). Interestingly, the *DET2* and *CYP 90A1* genes involved in the downstream biosynthetic pathway of steroidal alkaloids were significantly up-regulated, which was consistent with the accumulation of the peimine, peiminine, and hupehenine in our current study. Therefore, we speculated that *DET2* and *CYP 90A1* might play an important role in the accumulation of steroidal alkaloids in *F. hupehensis* in the I system. The significant positive correlation between gene expressions in the proposed biosynthetic pathway and the accumulation of these main steroidal alkaloids further validated our proposal.

Plant hormones and their signaling networks are involved in the biosynthesis and metabolic pathways of secondary metabolites (Tarkowská and Strnad, 2016; Kurepin et al., 2017; Thakur et al., 2019). MYC2 plays a key role in the JA signaling pathway, which regulated the biosynthesis of many alkaloids (Zhang et al., 2011; Thakur et al., 2019). Interestingly, in our study, 8 genes encoding MYC2 participating in the JA signaling pathway were down-regulated in the I system, which may play a crucial role in promoting the accumulation of steroidal alkaloids in *F. hupehensis*. Moreover, the other three hormone signaling pathways associated with cell enlargement, plant growth, fruit ripening, and cell division were also analyzed between these two cropping systems. The significantly higher expressions of the *SAUR, GH3*, and *AUX/IAA* genes in the I system might lead to the accumulation of steroidal alkaloids in *F. hupehensis.* (Zhao et al., 2018). However, the crosstalk between plant hormone signal transduction and the biosynthesis of steroidal alkaloids is complex, and further investigation is needed to elucidate the interactive functions of hormones in the accumulation of steroidal alkaloids in *F. hupehensis*.

Oxidative phosphorylation is of central importance to plant cells and plays an important role in electron transfer and energy supply in metabolic processes (Saraste, 1999; Braun, 2020).In our study, the expression levels of genes encoding NADH dehydrogenases (NDUS3, NDUS5A, NDUAS6, NDUAS12-1, and NDUAS12-2), COX6a, and ATP synthases (ATPSBM, ATPSGM, LEUING, VATPSE1-1, VATPSE1-2, ATP16PS, PMATP11, PMATP-1, PMATP-2, PMATP-3, PMATP-4, PMATP-5, PMATP-11, and PMATP4) were significantly up-regulated in the I system, which was consistent with the increase in peimine, peiminine, and hupehenine. Thus, we hypothesized that these enzymes involved in the oxidative phosphorylation pathway might play a key role in steroidal alkaloids accumulation and accelerate oxidative phosphorylation metabolism to release more energy for the synthesis of steroidal alkaloids.

CYPs are the largest enzyme family in plants (Zhao et al., 2018; Kumar et al., 2020; Kumar et al., 2021). CYP 710A and CYP 90B are reported to associate with the accumulation of steroidal alkaloids in *Fritillaria*. In this study, 14 genes encoding CYPs were up-regulated, whereas 30 genes were down-regulated, indicating that CYPs might play a complex role in steroidal alkaloid biosynthesis. ABC transporters have been reported to mobilize, transport, and regulate secondary metabolites in plants (Yazaki, 2006; Kang et al., 2011). In our study, 88 DEGs encoding ABC transporters were identified in *F. hupehensis*, however, their expressions were inconsistent in the I system. TFs are involved in the accumulation of secondary metabolites through complicated cross-interactions and signal crosstalk (Meraj et al., 2020). NAC, bHLH, MYB, MYC2, and WRKY are known to mediate the regulation of steroidal alkaloid biosynthesis in *Fritillaria*, as shown in previous studies (Zhao et al., 2018; Kumar et al., 2021; Sharma et al., 2021). In our study, most of the genes encoding MYB-related, GARP-G2-like, NAC, C3H, and HSF were up-regulated, whereas the bulk of genes encoding bHLH, AP2/ERF-ERF, C2H2, and MYB were down-regulated. These findings suggest that the genes mentioned above may play a pivotal role in the transportation and accumulation of steroidal alkaloids in *F. hupehensis*. However, the interaction among CYPs, ABC transporters, TFs and steroidal alkaloids biosynthesis are comprehensive and complex, and further verification is needed.

## Conclusion

**Figure 8:**
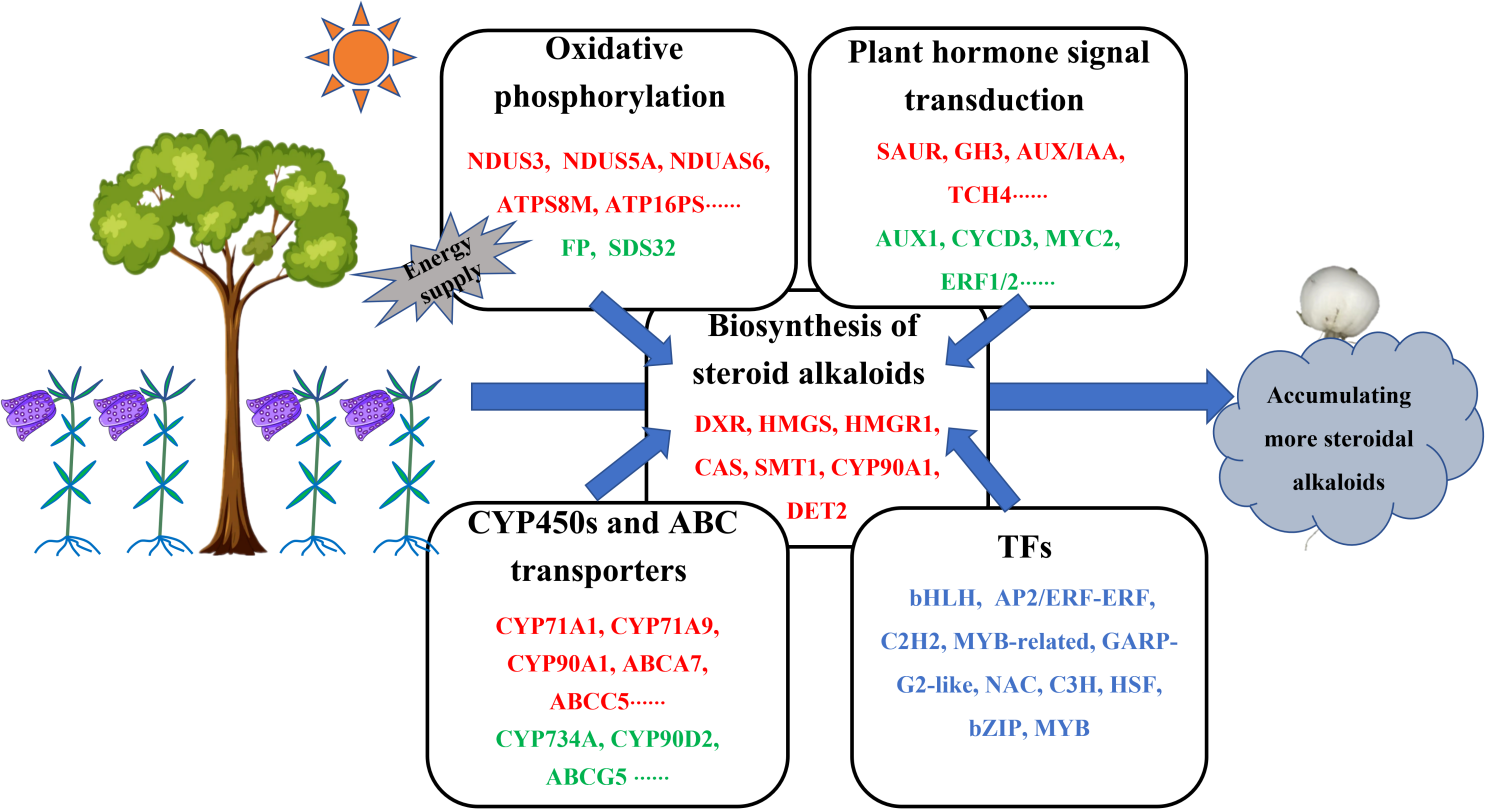
Proposed main molecular mechanisms of improving steroid alkaloids of *F. hupehensis* in response to the intercropping system. Up-regulated genes were denoted by red color, down-regulated genes were denoted by green color, both up-regulated and down-regulated genes were denoted by blue color.

This study demonstrated the roles and underlying mechanisms of the intercropping system, *F. hupehensis*-*M. officinalis* in regulating steroidal alkaloid biosynthesis in *F. hupehensis*. The results showed that 12 differentially expressed steroidal alkaloids were up-regulated in the intercropping system. A possible biosynthetic pathway for steroidal alkaloids in *F. hupehensis* was proposed based on transcriptome analysis. Genes involved in the proposed biosynthetic pathway were significantly positively correlated with the accumulation of peimine, peiminine, and hupehenine. Moreover, biological processes such as oxidative phosphorylation and plant hormone signal transduction might play crucial roles in the biosynthesis of steroidal alkaloids, especially oxidative phosphorylation, which may promote the accumulation of steroidal alkaloids in *F. hupehensis* by providing the necessary energy (**Figure 8**). In addition, CYPs, ABC transporters, and TFs were proposed to play an important role in the biosynthesis of steroidal alkaloids. This study provides a theoretical foundation and technical support for sustainable development of this ecological planting pattern. This study may be beneficial for improving the sustainability and production efficiency of *F. hupehensis*- *M. officinalis* intercropping ecosystems.

## Data availability statement

The datasets presented in this study can be found in online repositories. The names of the repository/repositories and accession number(s) can be found below: https://ngdc.cncb.ac.cn/gsub/, CRA007535.

## Author contributions

YD designed and performed the experiments, analyzed transcriptomic and metabolomic data, and wrote the manuscript; XL conceived this work and framed the experimental design; JW, FW and TT participated in plot selection, carried out the field trials, and collected the experimental samples; JY participated in the metabolomics and transcriptomics data analysis, participated in the revision of the manuscript; XG and ML guided the implementation of the field trials, and revised the manuscript; JG guided the experimental design, revised, and approved the final version of the manuscript. All authors contributed to this work and approved the manuscript.

## Funding

This work was supported by the Key Research and Development Plan Project of Hubei (2020BCA059), Hubei Technology Innovation Center for Agricultural Sciences - Key Research and Development Project of Science and Technology (2020-620-000-002-04), China Agriculture Research System (CARS-21), and the Forestry Science and Technology Project of Central Finance (2021TG16).

## Conflict of interest

The authors declare that the research was conducted in the absence of any commercial or financial relationships that could be construed as potential conflict of interest.

## Publisher’s note

All claims expressed in this article are solely those of the authors and do not necessarily represent those of their affiliated organizations, or those of the publisher, the editors and the reviewers. Any product that may be evaluated in this article, or claim that may be made by its manufacturer, is not guaranteed or endorsed by the publisher.
