# Supplementary material for: Transcriptomic and metabolic analyses reveal the potential mechanism of increasing steroidal alkaloids in Fritillaria hupehensis through intercropping with Magnolia officinalis
Source: Front Plant Sci. 2022 Oct 7;13:997868. doi: 10.3389/fpls.2022.997868 (PMC9585282; doi:10.3389/fpls.2022.997868)
Supplement: Supplementary file 1 [file DataSheet_1.docx]

Supplementary Material

# Supplementary Figures


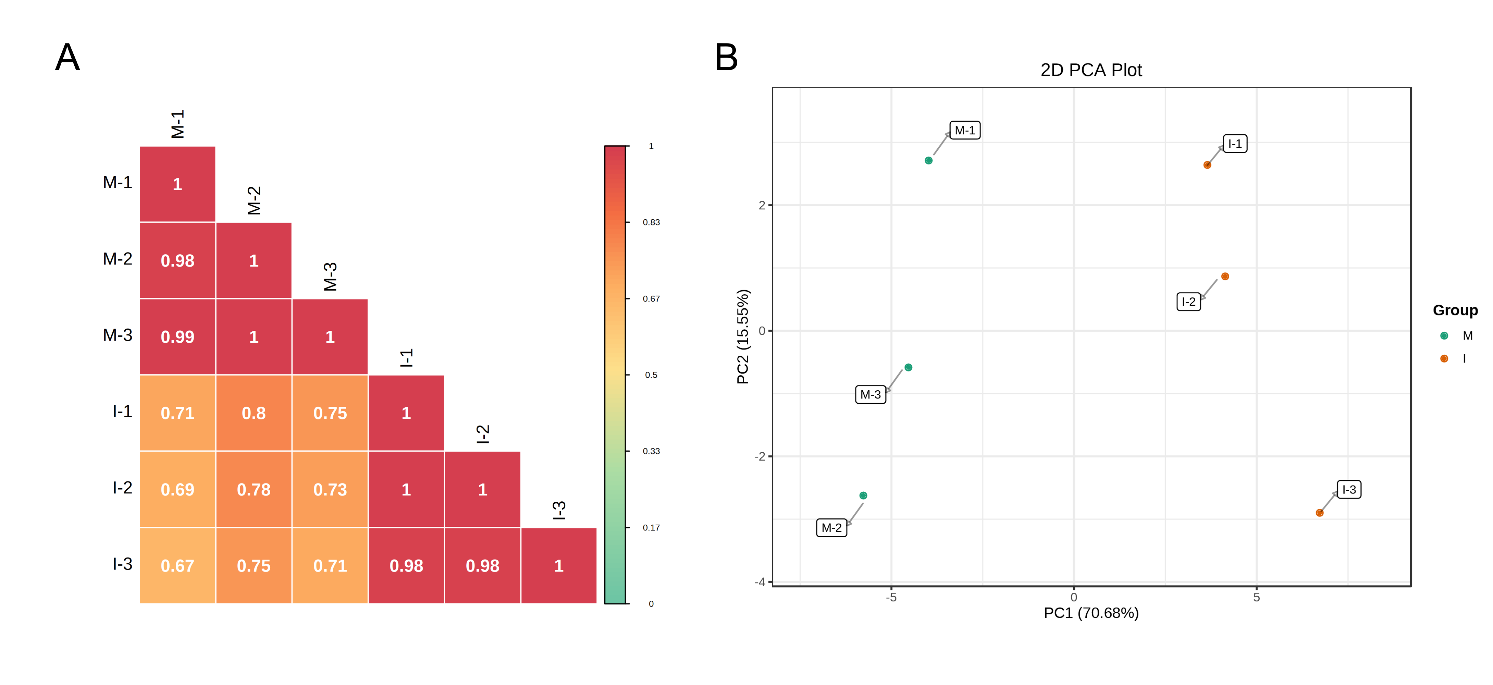


**Supplementary Figure 1** Correlation heat map analysis of *F. hupehensis* in the M and I systems based on UPLC-MS data (A) and Principal component analysis of UPLC-MS data (B).


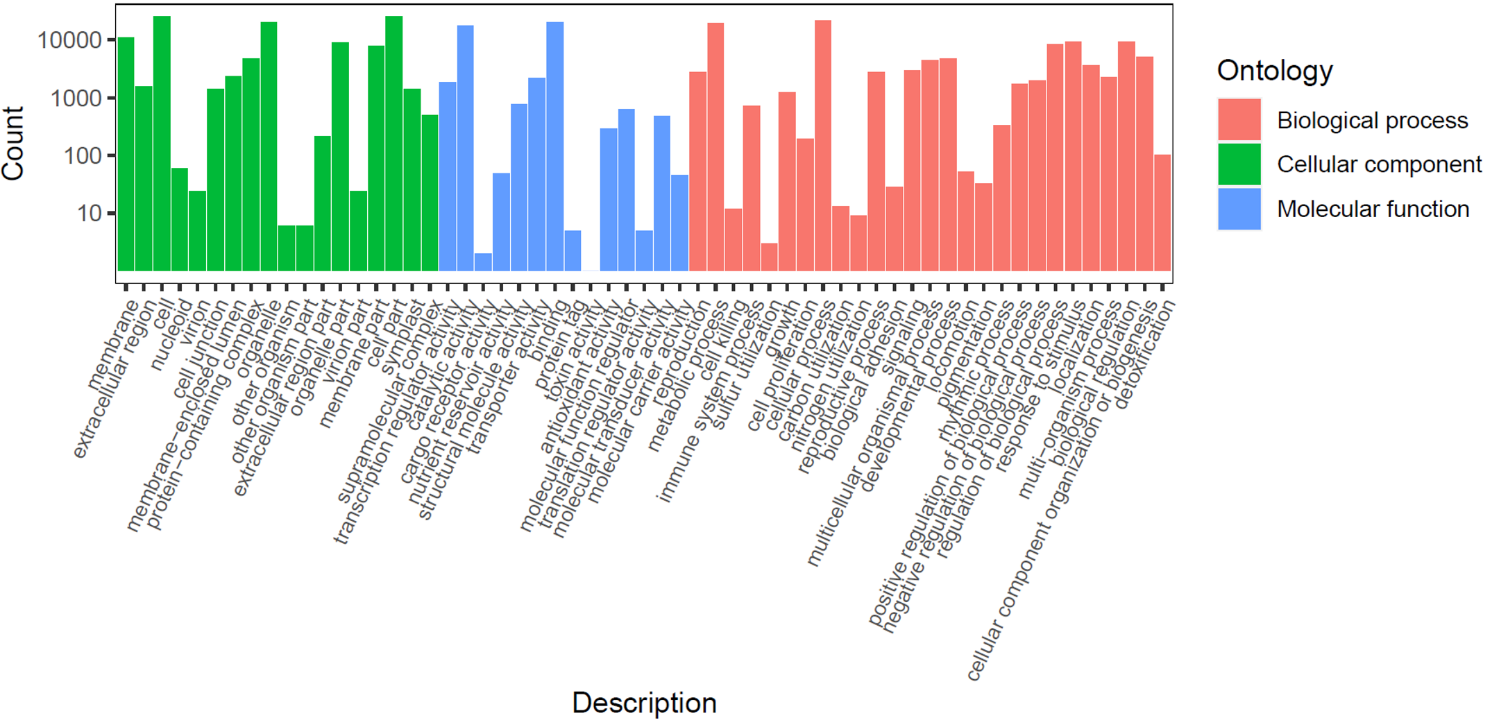


Supplementary Figure 2. GO annotation of *F. hupehensis* transcriptome sequencing data


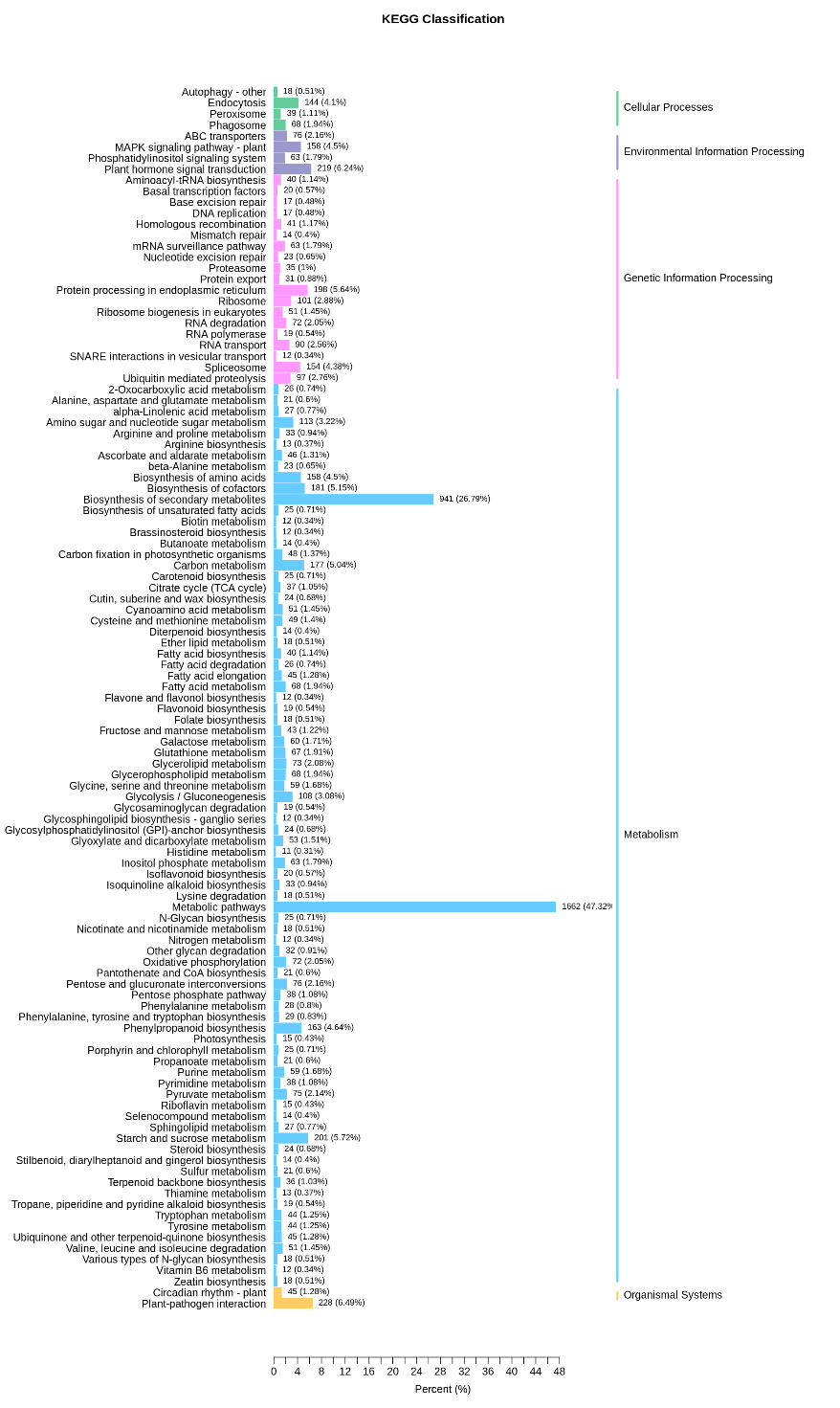
**Supplementary Figure 3.** KEGG pathway annotation of *F. hupehensis* transcriptome sequencing data.


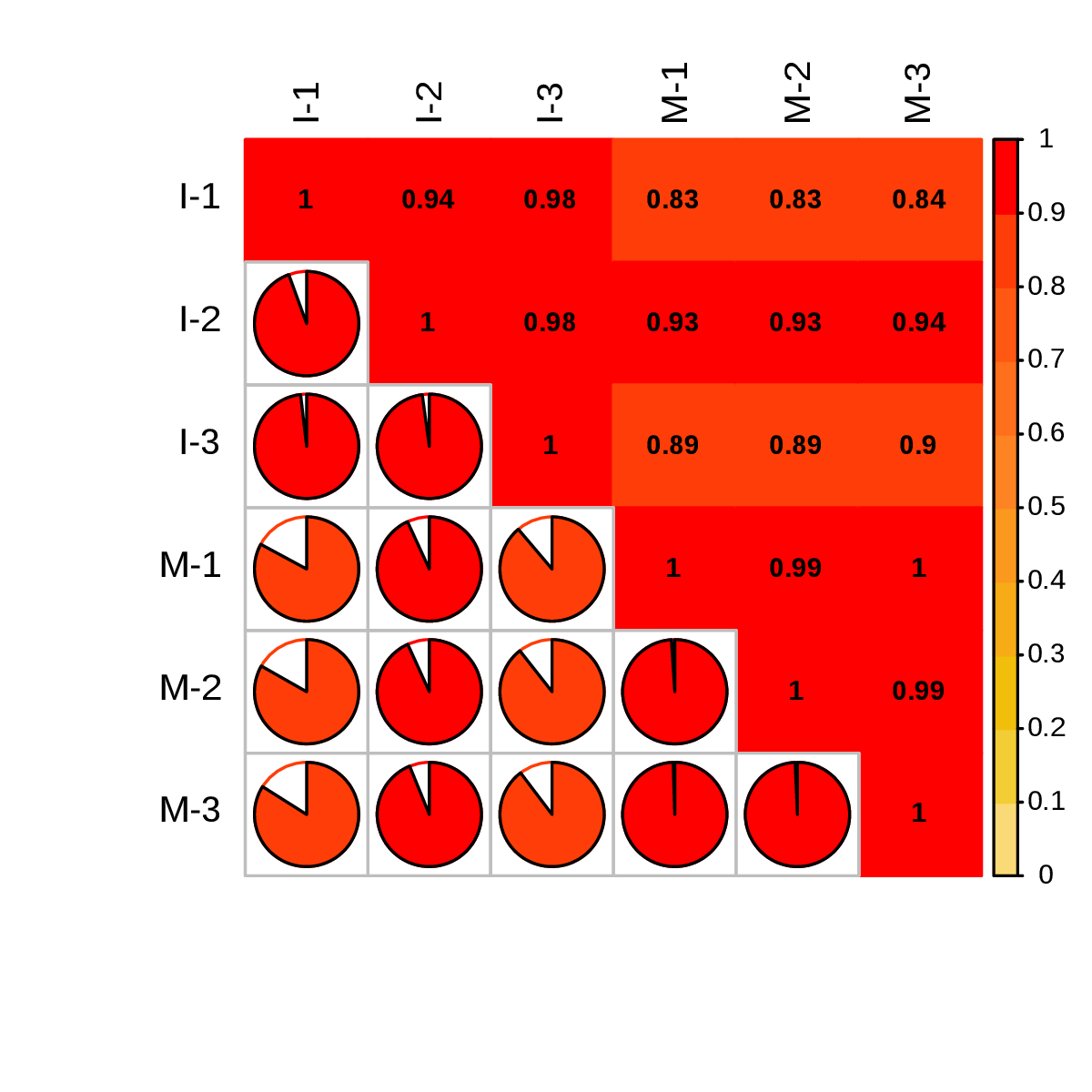
 **Supplementary Figure 4.** Correlation heat map analysis of *F. hupehensis* in the M and I systems based on gene expression profiles.
